# *Leishmania infantum* Amastigotes Trigger a Subpopulation of Human B Cells with an Immunoregulatory Phenotype

**DOI:** 10.1371/journal.pntd.0003543

**Published:** 2015-02-24

**Authors:** Guadalupe Andreani, Michel Ouellet, Rym Menasria, Alejandro Martin Gomez, Corinne Barat, Michel J. Tremblay

**Affiliations:** 1 Axe des Maladies Infectieuses et Immunitaires, Centre de recherche du Centre Hospitalier Universitaire (CHU) de Québec—pavillon CHUL, Québec, Canada; 2 Département de Microbiologie-Infectiologie et Immunologie, Faculté de médecine, Université Laval, Québec, Canada; University of Texas Medical Branch, UNITED STATES

## Abstract

Visceral leishmaniasis is caused by the protozoan parasites *Leishmania infantum* and *Leishmania donovani*. This infection is characterized by an uncontrolled parasitization of internal organs which, when left untreated, leads to death. Disease progression is linked with the type of immune response generated and a strong correlation was found between disease progression and serum levels of the immunosuppressive cytokine IL-10. Other studies have suggested a role for B cells in the pathology of this parasitic infection and the recent identification of a B-cell population in humans with regulatory functions, which secretes large amounts of IL-10 following activation, have sparked our interest in the context of visceral leishmaniasis. We report here that incubation of human B cells with *Leishmania infantum* amastigotes resulted in upregulation of multiple cell surface activation markers and a dose-dependent secretion of IL-10. Conditioned media from B cells incubated with *Leishmania infantum* amastigotes were shown to strongly inhibit CD4^+^ T-cell activation, proliferation and function (i.e. as monitored by TNF and IFNγ secretion). Blockade of IL-10 activity using a soluble IL-10 receptor restored only partially TNF and IFNγ production to control levels. The parasite-mediated IL-10 secretion was shown to rely on the activity of Syk, phosphatidylinositol-3 kinase and p38, as well as to require intracellular calcium mobilization. Cell sorting experiments allowed us to identify the IL-10-secreting B-cell subset (i.e. CD19^+^CD24^+^CD27^-^). In summary, exposure of human B cells to *Leishmania infantum* amastigotes triggers B cells with regulatory activities mediated in part by IL-10, which could favor parasite dissemination in the organism.

## Author Summary

Leishmaniasis is an infection caused by protozoan parasites of the genus *Leishmania* and is a significant neglected tropical disease, with 350 million people in 98 countries at risk of developing one of the forms of the disease. Visceral leishmaniasis is characterized by an uncontrolled parasitization of internal organs, which leads to death when left untreated. Disease progression is linked with the type of immune response generated and a strong correlation was found between disease progression and serum levels of the immunosuppressive cytokine IL-10. We demonstrate that a contact between human B cells with *Leishmania infantum* amastigotes resulted in upregulation of multiple cell surface activation markers and a dose-dependent secretion of IL-10. Conditioned media from B cells incubated with *Leishmania infantum* amastigotes were shown to strongly inhibit CD4^+^ T-cell activation, proliferation and function (i.e. TNF and IFNγ production). Blockade of IL-10 activity using a soluble IL-10 receptor restored to some degree TNF and IFNγ secretion. Cell sorting experiments allowed us to identify a major IL-10-secreting B cell subset characterized as CD24^+^ and CD27^-^. Exposure of human B cells to *Leishmania infantum* amastigotes thus triggers B cells with regulatory activities mediated in part by IL-10, which could promote parasite dissemination in the organism.

## Introduction

Leishmaniasis is an infection caused by protozoan parasites of the genus *Leishmania* and is one of the most significant neglected tropical diseases, with 350 million people in 98 countries worldwide at risk of developing one of the forms of the disease [1]. Visceral leishmaniasis (VL) is the most severe form of the disease and it represents nearly 40,000 deaths per year [1]. VL is characterized by an uncontrolled parasitization of organs, such as spleen, liver and bone marrow, and is caused by the species *Leishmania infantum* (*L*. *infantum*) (known as *L*. *chagasi* in South America) and *L*. *donovani*. All parasites of the genus *Leishmania* are obligate, intracellular protozoa that infect cells of the macrophage-dendritic cell lineage of their vertebrate hosts (primarily macrophages) [2,3]. The parasite exists under two distinct morphologic forms, i.e. either as motile promastigotes within the alimentary canal of their phlebotomine sandfly vector or as nonmotile amastigotes that reside within phagolysosomes of mammalian mononuclear phagocytes. Infection of the mammalian host is initiated when the female sandfly regurgitates infectious promastigotes during its blood meal. Promastigotes are quickly internalized by tissue phagocytes recruited to the site of infection. Following phagocytosis, promastigotes are engulfed in phagolysosomes, where they transform into the non-motile intracellular amastigotes. Thereafter, amastigotes replicate within acidic phagolysosomes, eventually lysing the cell and freeing themselves to interact with adjacent cells. According to a recent report, amastigotes could also be transferred directly to other target cells via LAMP-rich parasitophorous extrusions [4], exploiting an alternative mechanism of transmission that would minimize exposure to the immune system.

In the context of *L*. *major* infection in the BALB/c mouse model, the non-healing and disseminating form of leishmaniasis has been associated with a Th2 immune response, which is dominated by IL-4 (reviewed in [5]). Multiple reports suggest however that such polarized immune responses are not observed in humans and that elevated levels of interferon gamma (IFNγ) can be found in lesional tissues even during the acute phase of the disease [6–8]. Moreover, elevated levels of IL-10 in blood and tissues of VL patients are a better correlate of susceptibility than IL-4. The complexity of the immune response towards *Leishmania* and the extent of the cytokine network involved in humans is underscored by the wide variety of manifestations of the disease [9,10]. Studies in mice suggest that IFNγ-driven Th1 immune responses and IL-12 secretion play an important role for the control of the parasite and development of acquired immunity [11,12]. The overall immune response in the context of leishmaniasis requires the maintenance of a delicate balance between CD4^+^ and CD8^+^ T cells that are required for optimal cytokine secretion. These two cell populations play distinct but cooperative roles in disease resolution with CD4^+^ T cells being more involved in primary infection while CD8^+^ T cells are more important in secondary immune responses [13].

Evidence from mouse studies indicates that B-cell activation leads to disease exacerbation [14,15]. Furthermore, it has been shown that B-cell-deficient C57BL/6 mice are highly resistant to VL [16]. Very high titers of *Leishmania*-specific antibodies can be found in the serum of VL patients soon after infection but before the appearance of cellular immunological abnormalities [17,18]. This humoral response can persist for many years following treatment, thus suggesting a role for antibody-mediated immunity in protection against reinfection [19]. However, a strong correlation was found between seropositivity and progression to clinical diseases in healthy individual [20], suggesting a role for antibody production in disease pathogenesis. Indeed, some signs of B-cell dysfunctions are observed in human VL, including hypergammaglobulinemia and the presence of non-specific polyclonal and/or autoimmune antibodies [21–24]. In cutaneous leishmaniasis, polyclonal B-cell activation has been detected in response to *L*. *major* infection [25,26] and antibody production and antigen presentation by B cells have been shown to exacerbate disease also in *L*. *major* infection [27]. The importance of B cells for the development of a Th2 response and the susceptibility in BALB/c mice infected with *L*. *major* have been reported previously [27]. It has also been well established that the ability of B cells to direct the immune response in BALB/c mice toward a Th2 phenotype (associated with a non-healing disease) was dependent upon their capacity to present antigens to T cells rather than upon their production of specific IgG antibodies [27,28].

The regulatory cytokine IL-10 has repeatedly been implicated as an immunosuppressive factor in both human and experimental leishmaniasis (i.e. murine). For example, it has been reported that IL-10-deficient BALB/c and C57BL6 mice are highly resistant to *L*. *donovani* infection and blockade of IL-10 receptor in wild-type mice leads to control of the infection [29]. More recently, Deak and co-workers have shown that B cells are crucial for visceralization of *L*. *donovani* in the susceptible Balb/c mice model but that such effect is independent of IL-10 [14]. However the exact role of IL-10 secretion by B cells in the visceralization of *L*. *donovani* in humans still remains undefined. It has been shown that patients with an advanced state of disease display elevated levels of IL-10 mRNA in lesional tissues [6–8] and high amounts of IL-10 in serum [30–33]. Moreover, neutralization of IL-10 promotes clearance of the parasite in splenic aspirate cells from patients with VL [34]. Recent studies conducted in humans and mice have revealed that IL-10 can be produced by different cell types following *Leishmania* infection, including regulatory T cells (Tregs), Th1 cells [32,35–37], CD8^+^ T cells [38], natural killer cells, regulatory dendritic cells, macrophages [39], neutrophils [40] and B cells [14,41]. IL-10 acts as a multifactorial cytokine in human infectious diseases. By interfering with both innate and adaptative responses, it contributes to favourable conditions for the persistence of microbes and chronic infections. On the other hand, it prevents the development of immunopathological lesions that result from an exacerbated immune response to acute and chronic infections that can lead to deleterious tissue lesions [42]. Therefore, although high levels of IL-10 found in VL might help to diminish immunopathologies and tissue damage, the immunosuppressive effect of IL-10 might promote parasite replication, dissemination and disease progression.

Depending on the species and the disease studied but also on the triggering signals used, various populations of IL-10-secreting B cells with regulatory functions have been described. Yanaba and co-workers have described a rather unique murine CD1d^hi^CD5^+^ B-cell subset that is able to efficiently control T-cell-dependent inflammatory responses through IL-10 secretion [43]. Although a similar B-cell subset has been rarely described in humans, patients suffering from Chagas disease were shown to have a slightly higher frequency of CD1d^+^CD5^+^CD19^+^ B cells that produce IL-10 and an increased frequency of circulating CD1d^+^CD5^+^CD19^+^ B cells was correlated with inhibition of Th17 responses in tuberculosis patients [44]. A direct relationship between the CD1d^+^CD5^+^ regulatory B cells found in mice and humans must not necessarily be assumed however, as mice only express CD1d while humans can express other genes of the CD1 family (e.g. CD1a, b, c and e) [45].

The role of regulatory B cells in the development of autoimmune diseases and chronic infections in humans has been addressed previously and it was established that a CD24^hi^CD38^hi^ B-cell subset that secretes high amounts of IL-10 upon stimulation can regulate T-cell functions and is positively associated with renal graft acceptance [46,47]. Lastly, a human CD24^hi^CD27^+^ B-cell subpopulation was described as having regulatory properties upon stimulation by CPG/anti-CD40 and elevated frequencies of these cells were associated with various autoimmune diseases [48]. There seems to be no single marker to identify regulatory B cells and different subtypes of regulatory B cells might exist depending of various factors such as the disease and the nature of stimulation.

As already mentioned, B-cell responses and *IL-10* are involved in the pathogenesis of human VL. Given that colonization of secondary lymphoid organs occurs during VL and B cells are found at high concentrations in these tissues, it can be postulated that *Leishmania* parasites can interact with B cells. We investigated whether human B cells isolated do secrete IL-10 in response to *L*. *infantum* amastigotes. We assessed also the phenotypic characteristics of IL-10-secreting B cells and their capacity to modulate T-helper cell functions.

## Methods

### Ethical statement

The current study was approved by the Institutional Bioethics Committee (IBC) from the Centre Hospitalier Universitaire (CHU) de Québec—Pavillon CHUL. Clinical samples from tonsillar tissues were obtained from minor patients in accordance with the guidelines of the IBC and parents/guardians provided a written consent on behalf of all minor participants. Human primary CD4^+^ T cells were obtained from peripheral blood mononuclear cells (PBMCs) purified from the blood of healthy subjects in accordance with the guidelines of the IBC. A written ethics board-approved informed consent form was obtained from each donor for the CD4^+^ T cells.

### Isolation and purification of human B cells

Tonsillar tissues were obtained from 2- to 4-year old patients undergoing elective tonsillectomy (due to physiological reasons such as snoring, difficulty to swallow and obstructive sleep apnea) at the CHU de Québec—Pavillon CHUL. Briefly, tonsillar tissues were dissected into small pieces and resuspended in endotoxin-free phosphate-buffered saline (PBS) (Sigma, Oakville, ON) containing penicillin (100 U/ml) (Wisent, St-Bruno, QC), streptomycin (100 μg/ml) (Wisent, St-Bruno, QC), Fungizone/Amphotericin B (2.5 μg/ml) (Gibco, Burlington, ON) and Primocin (100 μg/ml) (InvivoGen, San Diego, CA). The tissue suspension was incubated with collagenase D (Roche Diagnostic, Montreal, QC) at a final concentration of 2 mg/ml for 45 min at 37°C. Next, partially digested tissue was processed with a GentleMacs device (Miltenyi Biotec inc. Auburn, CA) in a C tube using standard “program B”. DNAse I (Roche Diagnostic, Montreal, QC) was then added at a final concentration of 100 U/ml and the suspension was further incubated for 15 min at room temperature. Cell suspension was finally processed with the GentleMacs device (Miltenyi Biotec Inc., Auburn, CA) in a C tube using standard program “m_spleen_04”. The resulting cell suspension was diluted in a solution of PBS supplemented with 2mM EDTA and 0.5% BSA, filtered first through a 100-μm nylon mesh cell strainer (Partec, Swedesboro, NJ) and then through a 30-μm nylon mesh cell strainer (Partec), before being finally separated using a StemSep human B cell enrichment kit (StemCell Technologies, Vancouver, BC). Purity of human B cells was assessed by the expression of CD19 by flow cytometry, and populations with >95% of purity were used for the experiments. Isolated B cells (CD19^+^) were maintained at a density of 1 × 10^7^ cells/ml in RPMI-1640 medium (Gibco Life Technologies, Life Technologies, Burlington, ON) supplemented with 10% fetal bovine serum (FBS) (Wisent, St-Bruno, QC), penicillin (100 U/ml), streptomycin (100 μg/ml) and Primocin (100μg/ml). Various B-cell subsets were isolated with a BD influx high-speed cell sorter based on their expression of CD24, CD27 and CD38. Alternatively, CD27^-^ and CD27^+^ cells were separated using CD27 Microbeads (Miltenyi Biotec Inc.).

### Culture of *L*. *infantum* parasites

The *L*. *infantum* strain clone 1 (MHOM/MA/67/ ITMAP-263) used in this study and its maintenance as axenic amastigotes have been described previously [49]. Briefly, axenically-grown amastigote forms of *L*. *infantum* were maintained at 37°C under a 5% CO_2_ atmosphere by biweekly sub-passages in MAA/20 culture medium in 25 cm^2^ flasks. MAA/20 consists of modified medium 199 with Hank’s salts (Gibco, Burlington, ON), supplemented with 0.5% soybean tryptocasein (Pasteur Diagnostics, Marne la Coquette, France), 15 mM D-glucose, 5 mM L-glutamine, 4 mM NaHCO_3_, 0.023 mM bovine haemin and 25 mM HEPES (all purchased from Sigma, Oakville, ON) at a final pH of 6.5. Axenically grown amastigotes show morphological, biochemical and biological characteristics similar to those of *in vivo* amastigotes [49].

### Exposure of human B cells to *L*. *infantum* amastigotes

Purified human B cells were cultured at a final concentration of 1 x 10^7^/ml in RPMI-1640 culture medium containing L-glutamine supplemented with 10% FBS, penicillin (100 U/mL), streptomycin (100 μg/mL) and Primocin (100 μg/ml). Cells were incubated for 24 h with axenic amastigotes at a 3:1 ratio (unless otherwise indicated). Cells and/or cell-free supernatants were harvested for further analysis. In some experiments, cell culture inserts with a pore size of 1 μm (BD Falcon, BD Biosciences, Mississsauga, ON) were used to separate amastigotes from human B cells.

### Flow cytometry

Freshly isolated B cells were exposed to *L*. *infantum* amastigotes for 24 h after which cells were incubated for 20 min in PBS supplemented with 200 mM D-galactose (osmolarity was adjusted to 317 mOsml/l by reducing NaCl concentration accordingly) to detach the parasites from B cells. Control unexposed B cell cultures were also washed with D-galactose-supplemented PBS. Cells were then centrifuged at 300 *x g*, resuspended in the same solution and filtered through a 30-μm nylon mesh cell strainer (Miltenyi Biotec Inc., Auburn, CA), washed again and finally resuspended at 1 x 10^7^/ml in PBS containing 2 mM EDTA and 0.5% BSA (staining buffer). To monitor intracellular expression of IL-10, cells were incubated with GolgiPlug (BD Biosciences) during the last 5 h of stimulation along with ionomycin (1 μg/ml; Sigma, Oakville, ON) and *phorbol* 12-myristate 13-acetate (PMA) (50 ng/ml; Sigma, Oakville, ON). Viability of B cells was assessed by staining with the Fixable Viability Dye eFluor 780 in PBS/EDTA 2 mM, following manufacturer’s instructions (eBiosciences, San Diego, CA). Finally, cells were treated with 10% pooled human sera and 20% goat sera for 15 min at 4°C to block nonspecific binding sites and Fc receptors and cells were washed with cold staining buffer. Cells were centrifuged and resuspended at a final concentration of 1 × 10^7^ cells per 100 μl of cold staining buffer. For intracellular detection of IL-10, cells were stained with a combination of CD19-PE, CD27-FITC, CD24-PE and CD38-FITC monoclonal antibodies (mAbs) (BD Biosciences). Cells were washed, fixed and permeabilized using the Cytofix Cytoperm kit (BD Biosciences). Next, cells were stained with an APC-conjugated mouse anti-human IL-10 Ab (Miltenyi Biotec Inc.). B-cell activation markers following parasite exposure were evaluated by staining with anti-human CD25-PE, CD54-PE, CD80-PE, CD69-FITC, CD83-FITC, or CD86-FITC mAb (BD Biosciences). FMO controls including the Fixable Viability Dye and all the mAbs used were performed. Cells were analysed with a FACS Canto flow cytometer (BD Biosciences) and data were processed using FCS Express 4 (De Novo Software, Los Angeles, CA). For CD27 and CD24/CD38 cell sorting experiments, magnetically-enriched B cells stained as described above with corresponding fluorochrome-conjugated antibodies were sorted using a BD Influx high-speed cell sorter.

### Conditioned media preparation

Freshly purified B cells or sorted B cells (1 x 10^7^ cells/ml) were incubated for 24 h in the presence of *L*. *infantum* amastigotes at the previously indicated ratio. Control supernatants were prepared by leaving B cells untreated for 24 h in culture media. To evaluate the effect of soluble factors produced by *L*. *infantum* amastigotes, an additional control was prepared by incubating parasites in the same condition but in the absence of B cells. After incubation, supernatants were harvested and centrifuged at 2,000 *x g* for 10 min to eliminate cell debris and parasites. Finally, supernatants were filtered with a 0.22 μm sterile syringe filter, aliquoted and frozen at-80°C until used. All the cell-free supernatants were tested for IL-10 content using a commercial ELISA kit (see below for more details).

### Functional assays for human CD4^+^ T cells

Primary human CD4^+^ T cells were isolated from PBMCs using a magnetic Easysep CD4^+^ T-cell enrichment kit (Stemcell Technologies) and CD25^+^ T cells (both activated CD4^+^ T cells and Tregs) were depleted with a CD25 positive selection kit (Stemcell Technologies). For activation experiments, CD25^-^CD4^+^ T cells were activated with plate-bound mAbs directed against CD3 (clone OKT3; used at 5 μg/ml) and CD28 (clone 9.3; used at 2 μg/ml) for 48 h in the presence or absence of a 1:4 dilution of B-cell conditioned medium. Cells were then washed and stained with anti-human CD25-PE and CD69-FITC and analysed by flow cytometry. For proliferation experiments, resting CD4^+^ T cells were first stained with the carboxyfluorescein succinimidyl ester (CFSE) dye following manufacturer’s instructions (Invitrogen, Burlington, ON), activated as previously described for 5 days and analysed by flow cytometry. Production of TNF was evaluated by activating CD25^-^CD4^+^ T cells with plate-bound anti-CD3 (0.5 μg/ml) in the presence or absence of a 1:4 dilution of B-cell conditioned medium for 72 h. Cells were then washed, fixed and permeabilized using the Cytofix Cytoperm kit (BD Biosciences) and finally stained with a PE-conjugated anti-human TNF (BD Biosciences, Mississauga, ON) before being analysed by flow cytometry. In some cases, cell-free supernatants were pre-treated with a recombinant soluble IL-10 receptor alpha (ED_50_: 0.05–0.25 μg/ml in presence of 2 ng/ml of recombinant human IL-10) (R&D Systems, Minneapolis, MN) at a final concentration of 1 μg/ml. In all experiments, dead cells were excluded by 7-AAD staining as previously described [50].

### Quantification of IL-10

B cells were stimulated as previously described. Thereafter, cell-free supernatants were harvested and IL-10 secretion was quantified using a commercial BD OptEIA Human IL-10 ELISA set (BD Biosciences).

### qRT-PCR analysis

Total RNA was isolated using the illustra RNAspin Mini Kit (GE Healthcare, Mississauga, ON). RNA was reverse transcribed using Moloney-Murine Leukemia Virus (M-MLV) reverse transcriptase (Promega, Madison, WI) and expression levels of IL-10 transcripts were determined by quantitative PCR (qPCR) using SYBR Green master mix (Applied Biosystems, CA) on an ABI-PRISM 7500 Sequence Detector (Applied Biosystems). Each sample was run in triplicate and relative changes in IL-10 expression were calculated using the 2^ΔΔCt^ method [51]. This method was used once validation experiments showed that the efficiencies of the target and endogenous reference (18S) were comparable. The primers used for IL-10 were: Fwd: 5’-TTACCTGGAGGAGGTGATGC-3’ and Rev: 5’-GGCCTTGCTCTTGTTTTCAC-3’. These primers were designed using the coding sequence of human IL-10 (Accession NM_000572.2) and Primer3 web application (http://biotools.umassmed.edu/bioapps/primer3_www.cgi). The primers for 18S were: Fwd: 5’-TAGAGGGACAAGTGGCGTTC-3’ and Rev: 5’-CGCTGAGCCAGTCAGTGT-3’ [52].

### Signal transduction experiments

B cells were pre-treated for 45 min with the following pharmacological inhibitors: phosphatidylinositol 3-kinase (PI3K) inhibitor Wortmannin (0.625, 1.25, 2.5 and 5 nM) (InvivoGen, San Diego, CA), p38 MAP kinase inhibitor SB203580 (1.25, 2.5, 5 and 10 μM) (Invitrogen, Burlington, ON), Syk inhibitor IV (62.5, 125, 250, 500 and 1000 nM) (Calbiochem, EMD Millipore, Billerica, MA) and calcium chelator BAPTA/AM (1.25, 2.5, 5 and 10 μM) (Calbiochem). Dimethyl sulfoxide (DMSO) was used as a drug carrier control for each of the studied pharmacological inhibitors. After incubation with *L*. *infantum* amastigotes, cell-free supernatants were harvested and IL-10 was quantified as previously described. Cell viability was evaluated using 7-AAD staining (Biolegend, San Diego, CA) as previously described [50].

### TGF-β assay

Human B cells incubated or not with axenic amastigotes were cultured in FBS-containing RPMI-1640 culture medium or in serum-free XVIVO culture medium (Gibco Life Technologies). Cell-free supernatants were assayed for TGF-β using a commercial ELISA following the manufacturer’s instructions (R&D Systems). All supernatants were either assayed without treatment or following an acid/base treatment to activate latent TGF-β.

### Statistical analyses and data representation

Statistical analyses were performed using GraphPad Prism version 6 for Windows (GraphPad Software, La Jolla, CA). Two-tailed Student’s t-test was performed and a threshold of *p* < 0.05 was considered statistically significant.

## Results

### *L*. *infantum* amastigotes induce B-cell activation

**Fig 1:**
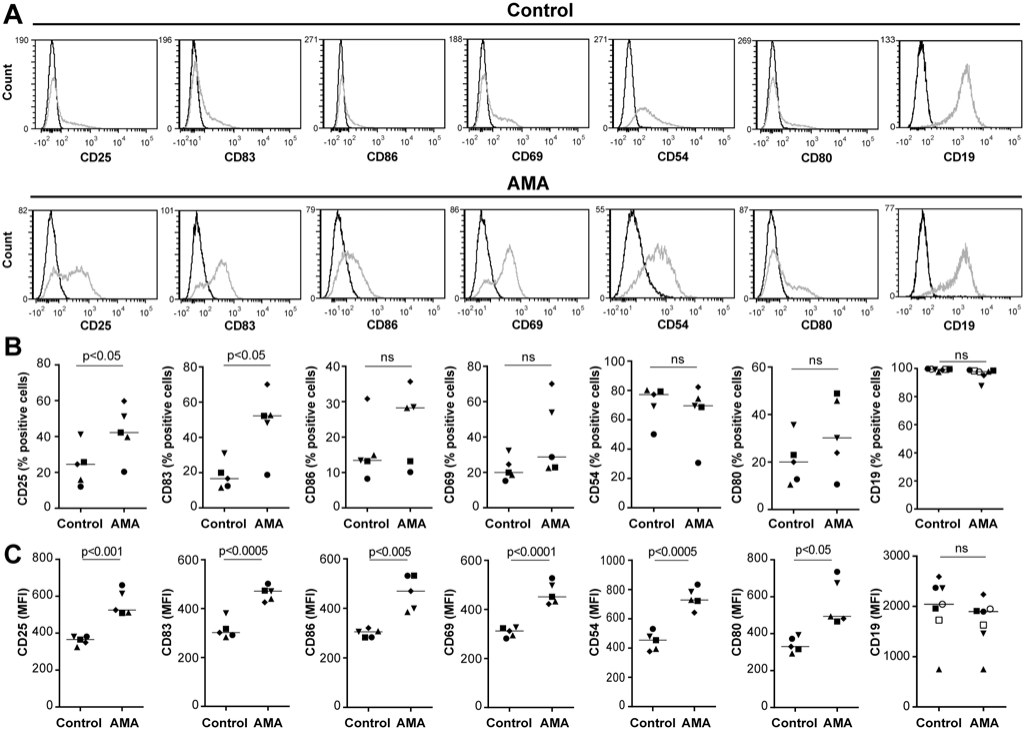
*L*. *infantum* amastigotes activate human B cells. Purified human B cells were either left alone (used as a control) or incubated overnight with *L*. *infantum* amastigotes (AMA) at a final parasite:host cell ratio of 3:1. Cells and the cell-parasite mixture were intensively washed with a galactose-modified PBS/EDTA solution and stained with the listed antibodies. Samples were read using a BD FACSCanto flow cytometer. A) Representative histograms displaying expression of each cell surface marker studied are shown in this panel. Dark lines on the histograms represent the fluorescence minus one (FMO) control for each sample. B) Results represent mean values of samples from 5 to 7 different healthy donors and are expressed as the percentages of positive cells for the indicated cell surface marker. C) Results represent mean values of samples from 5 to 7 different healthy donors and are expressed as the mean fluorescence intensities (MFI) for the indicated cell surface marker. *P* values were calculated by the two-tailed Student’s t-test (ns: not significant).

The potential outcome of an intimate contact between purified human B cells and *L*. *infantum* amastigotes was first assessed by monitoring surface expression of various cell activation markers by flow cytometry. Representative histograms of each activation marker tested are shown in Fig. 1A. Results depicted in Fig. 1B indicate that a statistically significant increase in the percentage of CD25- and CD83-expressing B cells is seen following an interaction with the parasite. The mean fluorescence intensities (MFI) for all studied activation markers (i.e. CD25, CD83, CD86, CD69, CD54 and CD80) were augmented in a statistically significant manner by a contact with parasites (Fig. 1C). On the other hand, the broadest lineage-specific surface marker for B cells, CD19, remains stable despite an incubation step with the parasite. Further experiments performed with cell culture inserts to separate amastigotes and B cells indicate that a physical contact between the two distinct entities is required to modulate B-cell activation (S1 Fig.).

### *Leishmania infantum* amastigotes induce IL-10 secretion by human B cells

**Fig 2:**
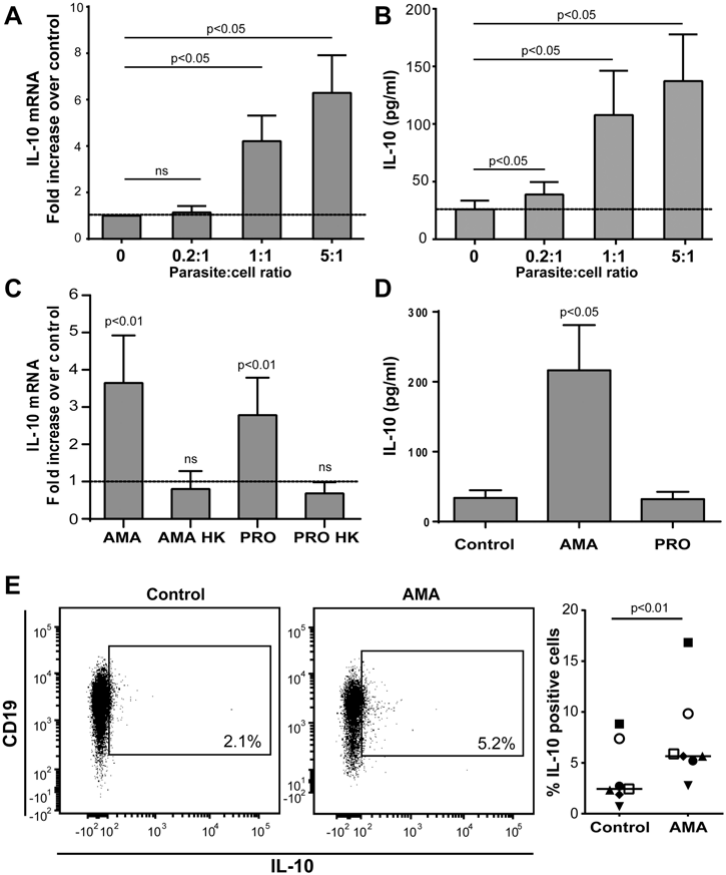
Incubation of human B cells with *L*. *infantum* amastigotes induces IL-10 production. A) Purified human B cells were either left alone (used as a control) or incubated overnight with *L*. *infantum* amastigotes at the listed parasite:host cell ratios. Next, IL-10 mRNA expression was measured by quantitative real-time PCR. Results are expressed as fold increase relative to cells left alone. *P* values were calculated by two-tailed Student’s t-test (n = 5). B) Cells were treated as in a panel A and IL-10 secretion was measured in cell-free supernatants by ELISA. Results are expressed as means +/- standard errors of the means (SEM) and *P* values were calculated by two-tailed Student’s t-test (n = 7). C) Purified human B cells were incubated overnight either with live *L*. *infantum* amastigotes, heat-killed (HK) *L*. *infantum* amastigotes, live *L*. *infantum* promastigotes (PRO), or heat-killed *L*. *infantum* promastigotes at a final parasite:host cell ratio of 3:1 before assessing IL-10 mRNA by quantitative real-time PCR. Results are expressed as fold increase relative to cells left alone. *P* values were calculated by two-tailed Student’s t-test (n = 5). D) Purified human B cells were incubated overnight either with live *L*. *infantum* amastigotes or live *L*. *infantum* promastigotes at a final parasite:host cell ratio of 3:1 before testing IL-10 production by ELISA. Results are expressed as means +/- SEM and *P* values were calculated by two-tailed Student’s t-test (n = 7). E) A representative dot plot of IL-10-expressing CD19^+^ B cells either left alone or incubated with *L*. *infantum* amastigotes (AMA) is shown on the left portion while percentages of IL-10-positive cells are displayed on the right portion of the panel. Results are expressed as means +/- SEM and *P* value is calculated by two-tailed Student’s t-test (n = 7).

As circulating IL-10 levels are elevated in VL patient, we assessed whether the parasite can drive IL-10 expression and secretion in human tonsillar B cells. A dose-dependent increase in IL-10 mRNA expression was seen in response to *L*. *infantum* amastigotes (Fig. 2A). Similar results were obtained when measuring IL-10 secretion in cell-free supernatants using a commercial ELISA kit (Fig. 2B). Interestingly, the parasite-dependent induction of IL-10 mRNA was not seen when using heat-killed parasites (Fig. 2C), thus suggesting that induction of IL-10 in B cells is an active mechanism mediated by live parasites. Although promastigotes can promote IL-10 mRNA synthesis at lower levels than amastigotes (Fig. 2C), they are unable to induce IL-10 at the protein level (Fig. 2D). Experiments performed with cell culture inserts to separate amastigotes and B cells indicate that a physical contact between human B cells and *L*. *infantum* amastigotes is necessary to achieve IL-10 secretion (S2 Fig.). The production of IL-10 was also determined by intracellular staining. The percentage of IL-10-expressing cells was higher in B cells exposed to the parasite compared to untreated controls (Fig. 2E), and were identified as CD19-expressing cells as previously described [46,48]. Importantly, human peripheral blood (circulating) B cells were also capable of secreting IL-10 upon incubation with the parasite and activation markers were also enhanced upon an exposure to *L*. *infantum* amastigotes (S3 Fig.). It has been previously described in mice that TGF-β is secreted by B cells with modulatory functions and is able to modulate CD4^+^ T-cell functions [53]. However, we were not able to detect TGF-β secretion or activation of latent TGF-β in B-cell culture media following exposure to parasites or in the supernatant of CD4^+^ T cells exposed to culture media from human B cells treated with amastigotes. Taken together these results demonstrate that a contact with *L*. *infantum* amastigotes activates human B cells and leads to secretion of T-cell suppressive factors such as IL-10. However, we have no evidence for a contribution of TGF-β in the suppressive phenotype.

### Parasite-mediated IL-10 production involves signalling via Syk, phosphatidylinositol 3-kinase, p38 and requires intracellular calcium

**Fig 3:**
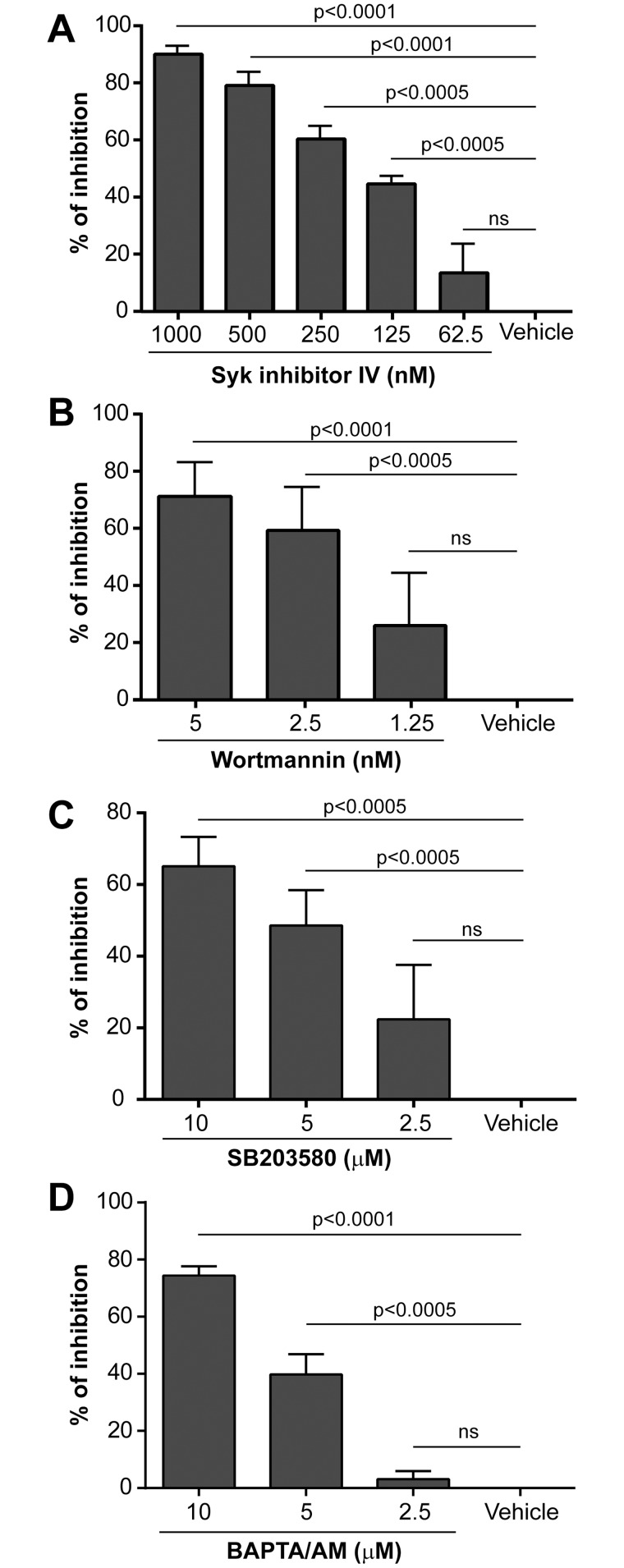
IL-10 secretion depends on p38, PI3K, Syk and calcium mobilization. Purified human B cells were either pretreated for 45 min with the vehicle (used as a control) or increasing concentrations of the listed specific inhibitors. Cells were then incubated overnight with *L*. *infantum* amastigotes at a final parasite:host cell ratio of 3:1. Finally, IL-10 production was quantified by ELISA. Results are expressed as means +/- SEM of the percentages of inhibition compared to the vehicle. Statistical significance was evaluated by two-tailed Student’s t-test (n = 5 to 7).

Secretion of IL-10 by macrophages and T cells has been shown to be mostly dependent on ERK and c-Maf signaling pathways although other second messengers such as p38 and NF-kappa B were involved as well [54–61]. In B cells, and particularly in murine regulatory B cells, IL-10 secretion has been suggested to rely on B-cell receptor (BCR)-derived signals leading to Syk activation and an increase in intracellular calcium but also on Toll-like receptor (TLR) expression [43,62,63]. Therefore, a panel of various signal transduction inhibitors was tested in order to shed light on the *signal transduction* pathways and molecular mechanisms that drive IL-10 production once human B cells are put in contact with *L*. *infantum* amastigotes. To this end, B cells were pre-treated with increasing concentrations of inhibitors directed against Btk (i.e. LFM-A13), ERK (i.e. PD98059), p38 (i.e. SB203580), protein kinase C (i.e. Rö318220), Syk (i.e. BAY 61–3606, also called Syk Inhibitor IV), and PI3K (i.e. Wortmannin) prior to exposure to the parasite. A dose-dependent inhibition of IL-10 secretion was observed only in the presence of inhibitors against Syk, PI3K and p38 (Fig. 3, panels A to C). No such decrease in the parasite-mediated induction of IL-10 was seen with compounds directed either against protein kinase C, ERK, or Btk. The role of intracellular calcium signalling was also investigated by pretreating B cells with increasing concentrations of the intracellular calcium chelator BAPTA/AM. A dose-dependent inhibition of IL-10 secretion in the presence of BAPTA/AM was detected (Fig. 3D), which is in line with a previous observation made with murine B cells [62]. Cell viability following drug treatment was evaluated by flow cytometry using 7-AAD staining of dead cells and no differences were observed between control samples and those treated with each drug at the various concentrations tested.

### Phenotypic characterization of IL-10-secreting B cells

**Fig 4:**
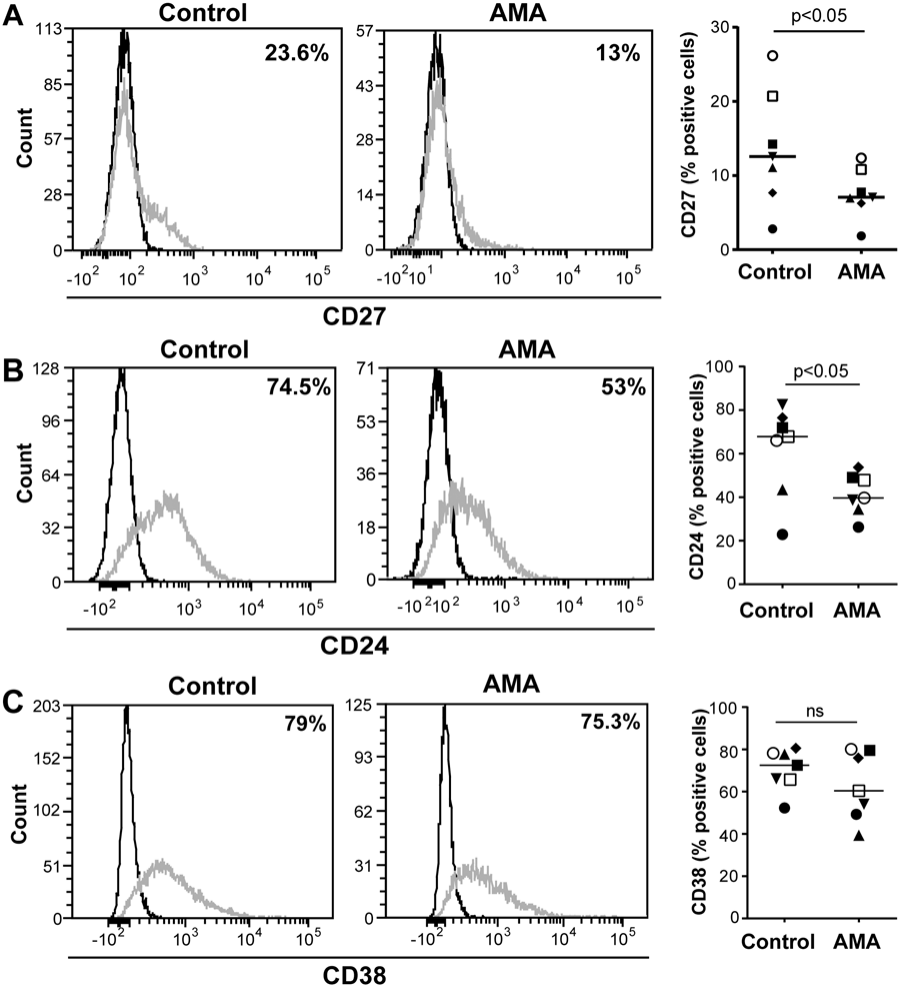
Surface markers of amastigote-stimulated B cells. Purified human B cells were incubated or not overnight with *L*. *infantum* amastigotes at a final parasite:host cell ratio of 3:1. Cells and the cell-parasite mixture were intensively washed with a galactose-modified PBS/EDTA solution and stained for CD27, CD24 and CD38. Dead cells were excluded by fixable viability dye staining. Representative histograms showing CD27 (panel A), CD24 (panel B) and CD38 expression (panel C) are shown on the left while percentages of positive cells are displayed on the right portion of the panel (n = 7). Dark lines on the histograms represent the FMO controls for each sample.

Although there is currently no cell surface or intracellular phenotypic marker or set of markers unique to IL-10-producing B cells, CD27 is a well-characterized marker for memory B cells which has been detected on some occasions in IL-10-secreting B cells [46,48,64]. It has also been established that transitional immature B cells (CD24^high^CD38^high^) are the main source of IL-10 produced by circulating B cells [46]. Moreover, it has been demonstrated that IL-10 is generated by CD24^high^CD27^+^ B cells and that an enhancement of CD24 and CD38 is seen in IL-10-expressing cells [48]. Therefore, we monitored surface expression of CD27, CD24 and CD38 on the total B-cell population after contact with *L*. *infantum* amastigotes. Data depicted in Fig. 4A demonstrate that the overall surface expression of CD27 diminished after an exposure to parasites compared to control cells. Similar results were obtained for CD24 (Fig. 4B), thus suggesting B-cell activation [65]. In contrast, CD38 was not affected upon exposure to the parasite (Fig. 4C).

**Fig 5:**
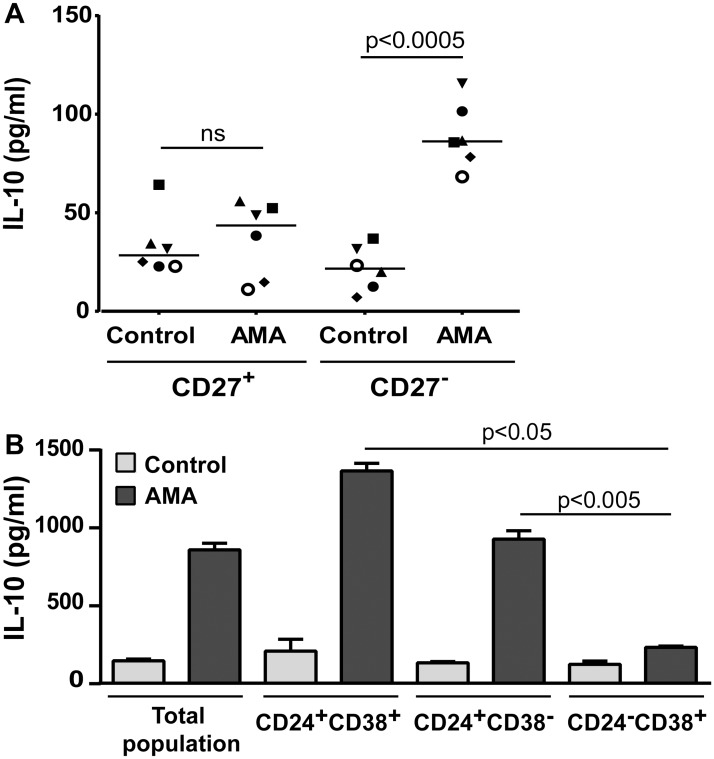
Characterization of the B-cell population(s) secreting IL-10 in response to parasites. A) Purified human B cells were separated in two distinct subpopulations (i.e. CD27^+^ and CD27^-^) and cells were incubated or not overnight with *L*. *infantum* amastigotes at a final parasite:host cell ratio of 3:1. Finally, IL-10 production was quantified by ELISA. Results are expressed as means +/- SEM and *P* values are calculated by two-tailed Student’s t-test (n = 6). B) Purified human B cells were either left unseparated or separated in three distinct subpopulations (i.e. CD24^+^CD38^+^, CD24^+^CD38^-^ and CD24^-^CD38^+^) by cell sorting. Next, cells were incubated or not overnight with *L*. *infantum* amastigotes at a 3:1 parasite to cell ratio. Finally, IL-10 production was quantified by ELISA. Representative results from one donor out of three are shown, results are expressed as means +/- SEM and *P* values are calculated by two-tailed Student’s t-test.

To determine the possible contribution of CD27 in the parasite-mediated B-cell response with respect to IL-10 secretion, experiments were performed using two distinct purified cell subpopulations (i.e. CD27^-^ and CD27^+^). Results showed that IL-10 is mainly secreted by the CD27^-^ subpopulation upon parasitic stimulation (Fig. 5A). To analyse the importance of CD24^+^ and/or CD38^+^ cells in response to *L*. *infantum* amastigotes, subsequent experiments were performed with three distinct purified B-cell subsets (i.e. CD24^+^CD38^+^, CD24^+^CD38^-^ and CD24^-^CD38^+^). The parasite-mediated induction of IL-10 is seen exclusively in the two CD24-expressing B-cell subsets irrespective of CD38 expression (i.e. CD24^+^CD38^+^ and CD24^+^CD38^-^) (Fig. 5B). These observations are in agreement with some previous studies, which were however not using a protozoan parasite such as *L*. *infantum* [46,48].

### Conditioned medium from parasite-treated B cells inhibits CD4^+^ T-cell functions

**Fig 6:**
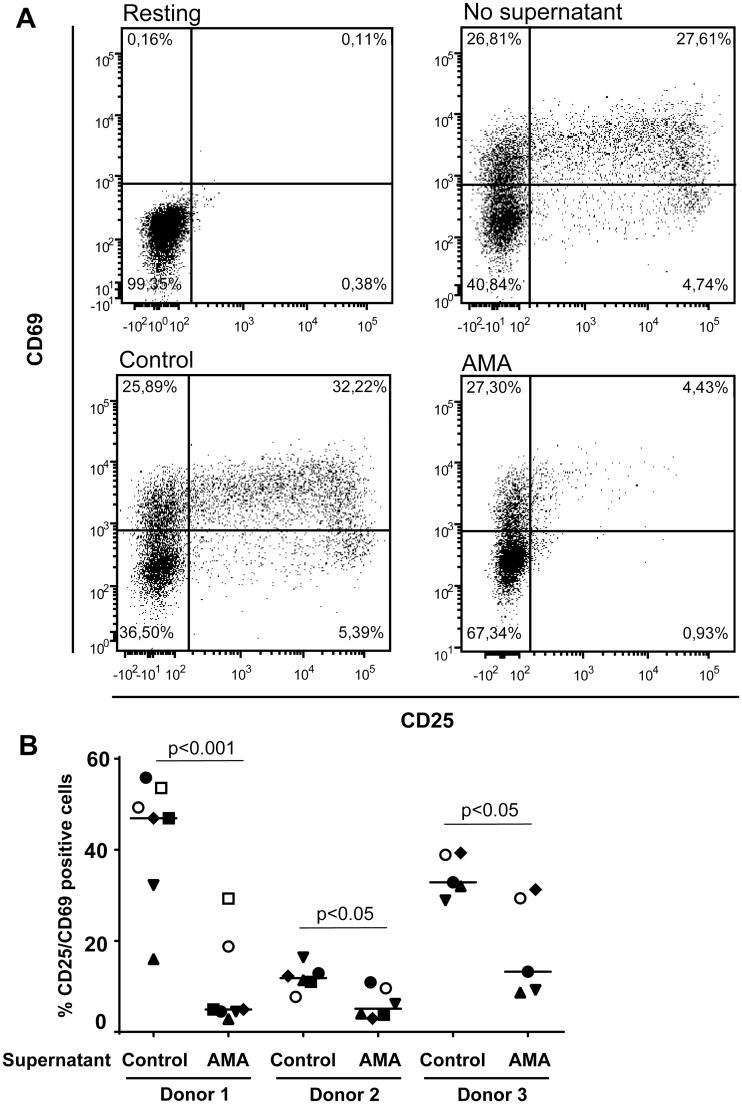
Conditioned medium from human B cells incubated with *L*. *infantum* amastigotes modulate CD4+ T-cell activation. Purified human CD25^-^CD4^+^ T cells were either left untreated (resting) or activated with plate-bound anti-CD3 and anti-CD28 antibodies. For activated cells, samples were either left untreated (no supernatant), incubated with cell-free supernatants from untreated B cells (control), or incubated with cell-free supernatants from B cells exposed to *L*. *infantum* amastigotes at a final parasite:host cell ratio of 3:1 (AMA). Seventy-two hours later, cells were analysed for cell surface expression of CD25 and CD69 by flow cytometry. Dead cells were excluded by 7-AAD staining. A) A representative dot plot is shown for each tested condition. B) Percentages of CD25^+^CD69^+^ Tcells for studies performed with CD25^-^CD4^+^ T cells from 3 different donors incubated with cell-free supernatants from 5 to 7 different B-cell donors. Statistical significance was evaluated by two-tailed Student’s t-test.

**Fig 7:**
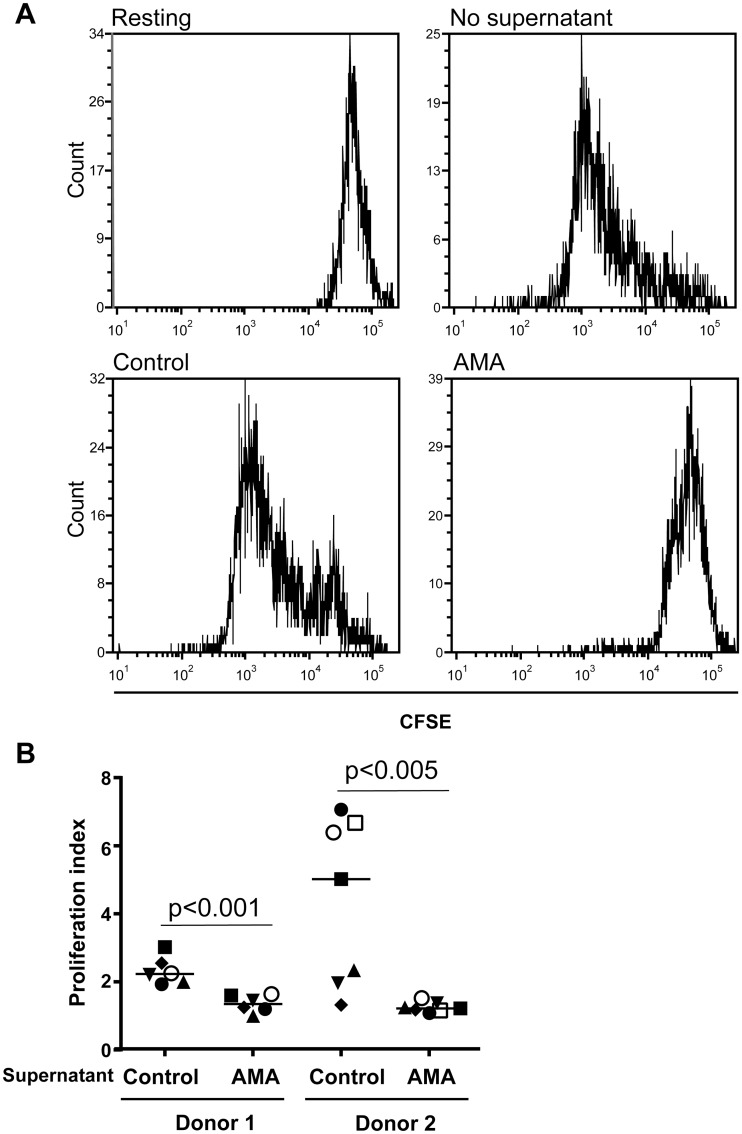
Conditioned medium from human B cells incubated with *L*. *infantum* amastigotes modulate CD4+ T-cell proliferation. Purified human CD25^-^CD4^+^ T cells were first labeled with CFSE and treated as described in Fig. 6. After 5 days, cell proliferation was assessed by CFSE dilution and dead cells were excluded by 7-AAD staining. A) A representative histogram is shown for each tested condition. B) Proliferation index for experiments performed with CD25^-^CD4^+^ T cells from 2 different donors incubated with cell-free supernatants from 6 or 7 different B-cell donors. Statistical significance was evaluated by two-tailed Student’s t-test.

Regulatory B cells have been shown previously to modulate CD4^+^ T-cell functions through different mechanisms involving soluble factors such as IL-10 [46,64]. Based on this information, we assessed whether conditioned medium from human B cells incubated with *L*. *infantum* amastigotes can modulate some basic functions of CD4^+^ T cells. It should be noted that CD25-expressing CD4^+^ T cells were depleted initially to remove both already activated cells and Tregs because it might represent a confounding factor. Results depicted in Fig. 6 indicate that soluble factors present in conditioned media from parasite-treated human B cells induce a statistically significant decrease in expression of the classical activation markers CD25 and CD69 on the surface of CD4^+^ T cells stimulated with plate-bound anti-CD3 and anti-CD28 antibodies (an experimental condition mimicking antigen presentation). A similar finding was made when measuring CD4^+^ T-cell proliferation with the CFSE dye (Fig. 7).

**Fig 8:**
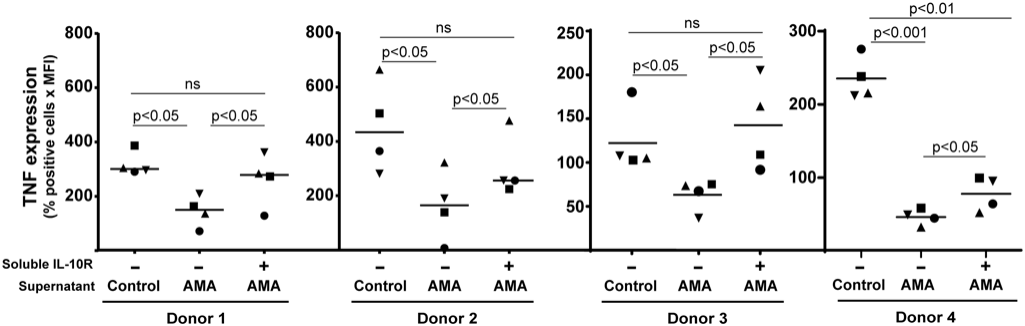
Parasite-mediated modulation of TNF secretion by CD4+ T-cell is partially dependent on IL-10. Purified human CD25^-^CD4^+^ T cells were activated for 72 h with plate-bound anti-CD3 and anti-CD28 antibodies in the presence of cell-free supernatants from B cells either left untreated (control) or incubated overnight with *L*. *infantum* amastigotes at a final parasite:host cell ratio of 3:1 (AMA). In some cases, cell-free supernatants from B cells incubated with parasites were treated with a soluble IL-10 receptor (1 μg/ml) before being used with activated CD25^-^CD4^+^ T cells. During the last 5 h of incubation, cells were further stimulated with PMA (50 ng/ml) and ionomycin (1 μg/ml) and Golgiplug™ was added (1 μl per 1 x 10^6^ cells). Finally, cells were fixed, permeabilized and stained for intracellular TNF before being analysed by flow cytometry. Dead cells were excluded by 7-AAD staining. Results are expressed as the percentages of TNF^+^ cells multiply by the mean fluorescence intensities (MFI). Data shown are the results from 4 different CD25^-^CD4^+^ T-cell donors incubated with cell-free supernatants from 4 different B-cell donors. Statistical significance was evaluated by two-tailed Student’s t-test.

**Fig 9:**
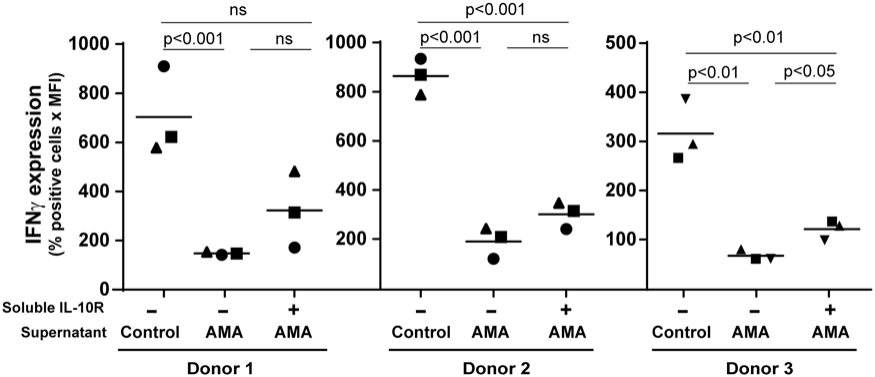
Parasite-mediated modulation of IFNγ secretion by CD4+ T-cell relies in part on IL-10. Purified human CD25^-^CD4^+^ T cells were activated for 72 h with plate-bound anti-CD3 and anti-CD28 antibodies in the presence of cell-free supernatants from B cells either left untreated (control) or incubated overnight with *L*. *infantum* amastigotes at a final parasite:host cell ratio of 3:1 (AMA). In some cases, cell-free supernatants from B cells incubated with parasites were treated with a soluble IL-10 receptor (1 μg/ml) before being used with activated CD25^-^CD4^+^ T cells. During the last 5 h of incubation, cells were further stimulated with PMA (50 ng/ml) and ionomycin (1 μg/ml) and Golgiplug™ was added (1 μl per 1 x 10^6^ cells). Finally, cells were fixed, permeabilized and stained for intracellular IFNγ before being analysed by flow cytometry. Dead cells were excluded by 7-AAD staining. Results are expressed as the percentages of TNF-positive cells multiply by the mean fluorescence intensities (MFI). Data shown are the results from 3 different CD25^-^CD4^+^ T-cell donors incubated with cell-free supernatants from 3 different B-cell donors. Statistical significance was evaluated by two-tailed Student’s t-test.

Secretion of TNF by activated CD4^+^ T cells is a well-recognized marker of their pro-inflammatory function [66,67], and it was shown to be modulated by regulatory B cells in both humans and mice [46,48]. We thus assessed intracellular TNF expression in CD4^+^ T cells treated with conditioned media from parasite-treated human B cells. A statistically significant diminution in the percentage of TNF-producing CD4^+^ T cells was seen following incubation with conditioned media from human B cells incubated with *L*. *infantum* amastigotes (Fig. 8), which was partially but not completely inhibited in the presence of a blocking agent (i.e. neutralizing soluble IL-10 receptor). Given the importance of IFNγ in the immune response directed against *Leishmania* parasites, we also performed intracellular staining for this cytokine. We found that cell-free supernatants from human B cells incubated with *L*. *infantum* amastigotes mediate production of IFNγ in CD4^+^ T cells, a process which was again not totally restored in presence of the neutralizing soluble IL-10 receptor (Fig. 9).

It was previously shown that inhibition of CD4^+^ T-cell proliferation was partially mediated by IL-10 [64]. However we were unable to restore CD4^+^ T-cell proliferation by using the neutralizing soluble IL-10 receptor. Altogether our observations suggest a partial involvement of IL-10 in the parasite-mediated modulatory effect on human CD4^+^ T cells.

## Discussion

The biological contribution of IL-10 for the visceralization of *L*. *infantum* in mice has been well established [29,68–70]. Moreover, IL-10 levels correlate with disease progression in humans [6,71] and dogs [72]. Finally, the importance of B cells as a source of IL-10 in the susceptible Balb/c murine model of *L*. *major* infection has been already described [41] and their potential to regulate T-cell responses was described in mice infected with *L*. *donovani* [15]. However, while Deak and colleagues have recently demonstrated the role of B cells in the visceralisation of *Leishmania* in a murine model of VL, they have shown that this effect is not relying on IL-10 [14]. In the spleen of VL patients, IL-10 mRNA was mostly associated with T lymphocytes and a reduced B/T ratio was observed compared to exposed controls. This would suggest a minor role for B cells in the elevated plasma IL-10 levels. However, our results in Fig. 2 (panels C and D) with B cells exposed to *L*. *infantum* promastigotes suggest that IL-10 mRNA levels are not necessarily correlated to IL-10 cytokine production. The direct involvement of human B cells to the elevated levels of plasma IL-10 following *L*. *infantum* infection and their role in the visceralization of *L*. *infantum* in humans still remains unclear. In the current study, we first demonstrate that *in vitro* incubation of *L*. *infantum* amastigotes with purified human B cells increases expression of numerous activation markers (Fig. 1) and the parasite-directed effect on B-cell activation requires an intimate contact between the two distinct entities (S1 Fig.). This is in agreement with the demonstrated B-cell activation in patients with localized cutaneous leishmaniasis [73].

We provide evidence that *L*. *infantum* amastigotes induce a dose-dependent increase in IL-10 mRNA expression and cytokine secretion (Fig. 2). An enhancement of IL-10 production was also observed when using peripheral blood B cells (S3 Fig.). The parasite-mediated production of IL-10 by B cells was not seen with parasites under the flagellated promastigote form, paraformaldehyde-killed amastigotes (Fig. 2) and when abrogating an intimate contact between parasites and B cells (S2 Fig.), therefore suggesting that a direct contact of live parasites under the nonflagellated amastigote stage (normally found in the macrophage phagolysosomal compartment of the mammalian host) is required to mediate the observed phenomenon. Intracellular staining by flow cytometry revealed that IL-10-expressing cells correspond to the CD19 compartment, as it has been previously described for other types of stimulation [46,48]. Our results therefore suggest that while B cell-derived IL-10 driven by amastigotes could contribute significantly to the elevated levels of IL-10 observed in VL patients, this cytokine is probably not the sole factor involved in the amastigote-induced B-cell regulatory activity.

A very close physical contact between human B cells and *L*. *infantum* amastigotes was observed under the microscope and the use of a galactose-modified PBS/EDTA solution was required to efficiently separate the two distinct entities and obtain single-cell suspensions prior to flow cytometry analyses. The ability of galactose to take apart B cells and parasites suggests the involvement of a galactose-specific lectin. Multiple lectins can transduce signals in response to ligand binding and some of them have been shown to bear one or multiple intracellular tyrosine-based activation motifs (ITAM) that can signal through Syk, intracellular calcium and PI3K to activate endocytosis, cell proliferation and gene transcription [74–77]. The protozoan parasite *Leishmania* has been shown to be bound by various lectins, including plant lectins as well as mammalian C-type lectins and galectins [78–84]. Lectin ligands on *Leishmania* have been shown to be both species- and stage-specific, which complicate their identification and the study of their functionality [81,82,84]. *Leishmania* can also trigger TLR signaling, mostly via TLR2, TLR4 and TLR9 [40,68,85–89]. In mice, TLR signaling through Myd88 was shown to be essential for the clearance of the pathogen [90]. It has been show that TLR4 and TLR9 agonists induce IL-10 in murine B cells, which can modulate T-cell responses [43,48]. Altogether, these observations suggest that *Leishmania* can induce activation of regulatory B cells via a TLR-dependent pathway. Experiments aimed at evaluating the putative contribution of TLR- and Myd88-mediated signal transduction pathways in the parasite-induced IL-10 secretion were unsuccessful due to technical limitations related to low transfection efficiency in primary human B cells and unstable or inefficient commercial inhibitory peptides. However, we could not detect any parasite-induced secretion of type-I IFN in human B cells, as monitored with the reporter cell line HEK-Blue™ IFN-alpha/beta using various parasites to cell ratios and different incubation periods. As type-I IFN production is a hallmark output of endosomal TLR signaling (i.e. TLR3, 7, 8 and 9) [91,92] and as human tonsillar B cells do not express TLR4 and do not respond to LPS [93,94], we can at least propose that the parasite-triggered IL-10 secretion does not rely on these receptors. Production of IL-10 by regulatory B cells in peripheral blood was also shown to be dependent on BCR, ERK and intracellular calcium [43,62]. We could not observe any modulatory effect of the highly specific ERK inhibitor PD98059 on IL-10 secretion following incubation with *L*. *infantum* amastigotes. Similarly, the BTK inhibitor LFM-A13 and the PKC inhibitor Rö318220 did not affect the parasite-mediated IL-10 secretion. ERK, BTK and PKC are known to be important for signal transduction via the BCR [95–98]. Our experiments instead suggest that the parasite-mediated IL-10 secretion in human B cells is dependent on Syk, PI3K, p38 and intracellular calcium (Fig. 3), which would again point towards a C-type lectin-mediated signal transduction pathway. Additional studies are required to identify the ligand(s) of amastigotes on the surface of human B cells.

Although regulatory B cells and IL-10 secretion by B cells have been observed in humans [46,48,99], the precise phenotype of such cells is still elusive. It has been previously shown that stimulation of human B cells with CpG and anti-IgG drives IL-10 secretion by memory B cells (IgD^+^CD27^+^) after a short period of treatment (i.e. 5 h), whereas naïve B cells were shown to produce IL-10 following a longer period of stimulation with the same agonists (i.e. 48 h) [64]. The controversy surrounding the phenotypic characterization of IL-10-producing B cells is further supported by the previous findings made by Iwata an colleagues who have shown that the CD27^+^ subpopulation can respond to CpG and CD40L by expressing IL-10 [48] and Blair and co-workers who have established that IL-10-producing B cells following stimulation with CD40L correspond to the CD27^-^ subset [46]. Our results indicate that CD27 expression decreases in the whole population of human B cells following treatment with *L*. *infantum* amastigotes (Fig. 4A). This might suggest a decrease of the memory B-cell population, as it was observed for other pathologies including HIV-1 infection [100,101].

The transitional immature B-cell subset (CD24^high^CD38^high^) has been proposed as the main producer of IL-10 by the B-cell population [46]. However, in another study, the IL-10-secreting cells were predominantly found in the CD24^high^CD27^+^ B-cell population [48]. Considering that a consensus about CD24 expression seemed to emerge as a hallmark for IL-10-secreting human B cells, we monitored expression of this surface marker upon incubation of human B cells with *L*. *infantum* amastigotes. We report herein that the parasite leads to a significant reduction in the expression of CD24 (Fig. 4B), indicative of B-cell activation [102,103], with no effect on CD38 (Fig. 4C). Altogether, these results suggest that the phenotype of IL-10-secreting B cells following incubation with *L*. *infantum* is different from those that were described previously. In a related set of experiments, we studied different purified B-cell subsets (i.e. CD24^+^, CD27^+^ and CD38^+^) and surprisingly, only the CD27-negative B-cell subpopulation responded to the parasite by producing IL-10 (Fig. 5A). Furthermore, we were able to reveal the importance of CD24^+^ cells, but not CD38^+^, for the secretion of IL-10 by B cells in response to *L*. *infantum* amastigotes (Fig. 5B). This suggests a quite different IL-10-secreting B-cell subset from those that were previously described both in *human in vitro* and *murine in vivo* models [41,43,46,48]. While it is assumed that phenotypic characteristics of IL-10-producer cells might differ according to the stimuli to which they are exposed, this CD24^+^CD27^-^ human B-cell subpopulation seems to respond to *L*. *infantum* amastigotes by secreting high levels of IL-10. Given that our experimental procedures involved a short incubation period between human B cells and parasites (i.e. 24 h) without involvement of CD40 signaling, we postulate that such a CD40-independent activation of B cells would arise as part of an innate response, which could be exploited by the parasite.

Data from additional experiments suggest that soluble factors secreted by human B cells exposed to *L*. *infantum* amastigotes can inhibit activation and proliferation of CD25^-^CD4^+^ T cells (Figs. 6 and 7), which corroborates a previous work using human B cells treated with a combination of a TLR9 agonist (i.e. CpG-B) and anti-Ig antibodies [64]. Regulatory B cells are characterized by their ability to modulate different functions of CD4^+^ T cells including activation, proliferation and production of pro-inflammatory cytokines such as TNF and IFNγ. Such cells can also significantly regulate some biological functions of monocytes and dendritic cells [46,48,64,104]. Among their described mechanisms of action, IL-10 remains the major soluble factor involved but modulation of cell function though TGF-β has also been described in the murine model [53]. A more direct interaction may also be at play since Blair *et al*. have reported that CD80 and CD86 interactions between B cells and CD4^+^ T cells act synergistically with B cell-derived IL-10 to suppress CD4^+^ T-cell cytokine production [46]. Although it was previously shown that inhibition of CD4^+^ T-cell proliferation was mediated by IL-10 [64], we did not see a significant restoration of cell proliferation by neutralizing IL-10 in the cell supernatant via addition of a soluble IL-10 receptor. These results lead us to propose that impairment of CD25^-^CD4^+^ T-cell proliferation by soluble factors secreted by human B cells incubated with *L*. *infantum* amastigotes is independent of IL-10.

Production of TNF by CD4^+^ T cells following antigenic stimulation can be used as a functional marker [66,67]. It was established that conditioned media from CPG/anti-Ig-treated B cells could inhibit TNF production by antigen-stimulated CD4^+^ T cells but that this effect was not relying on IL-10 [48]. Interestingly, TNF secretion by monocytes was demonstrated to be affected by conditioned media from B cells in an IL-10-dependent manner [48]. In our hands, when CD25^-^CD4^+^ T cells were activated in the presence of conditioned media from B cells exposed to *L*. *infantum* amastigotes, we observed a significant decrease in TNF production, which was partially reverted when adding a soluble IL-10 receptor (Fig. 8). Interestingly, similar observations were made when IFNγ was used as readout (Fig. 9). It can be hypothesized that modulation of CD4^+^ T-cell functions exerted by conditioned media from parasite-treated human B cells is due to a multifactorial process that includes IL-10 and some unknown factor(s). The parasite-directed enhancement in CD80 and CD86 expression in human B cells (Fig. 1) might be involved in modulating CD4^+^ T-cell functions [46], however we were unable to evaluate this possibility due to technical limitations related to parasite growth.

The systemic dissemination of pathogens such as *Staphylococcus aureus*, *Porphyromonas gingivalis*, *Mycobacterium tuberculosis* and Herpesviruses has been linked with their ability to induce IL-10 secretion in immune cells [105–109]. More relevant to the present study, IL-10 secretion is crucial for the visceralization of *Leishmania* parasites in mice [29,110–112] and increased levels of IL-10 are found in the serum of patients suffering from VL [23,31,71,73]. The contribution of B cells to the production of IL-10 in response to *Leishmania* has also been described in the mice model [14,15,41]. However, VL in humans has been associated with a reduced B/T ratio in the spleen and elevated IL-10 mRNA mainly associated with T lymphocytes [32]. Herein, we report that *L*. *infantum* amastigotes can induce secretion of IL-10 and other(s) unknown suppressive factors in a specific subset of human B cells (i.e. CD19^+^CD24^+^CD27^-^) that can modulate CD4^+^ T-cell functions. Further studies are warranted to determine the clinical importance of this phenomenon and whether induction of B regulatory functions by *L*. *infantum* amastigotes is part of an immune evasion strategy that would allow the dissemination and visceralization of this parasite.

## Supporting Information

S1 Fig Parasite-directed B-cell activation necessitates a direct contact. Purified human tonsillar B cells were either left untreated or incubated overnight with *L*. *infantum* amastigotes (AMA) at a final parasite:host cell ratio of 3:1. In some wells, cell culture inserts were used to separate parasites and B cells. Cells and the cell-parasite mixture were then intensively washed with a galactose-modified PBS/EDTA solution and stained with anti-CD25 or anti-CD83 antibodies. Samples were read using a BD FACSCanto flow cytometer. Results represent individual and mean values of samples from 4 different healthy donors and are expressed as the percentages of CD25^+^ (left panel) or CD83^+^ cells (right panel). *P* values are calculated by two-tailed Student’s t-test (ns: not significant).
(TIF)

S2 Fig Direct contact between *L*. *infantum* amastigotes and B cells is required for IL-10 induction. Purified human tonsillar B cells were either left untreated or incubated overnight with *L*. *infantum* amastigotes (AMA) at a final parasite:host cell ratio of 3:1. In some wells, cell culture inserts were used to separate parasites and B cells. IL-10 secretion was measured in cell-free supernatants by ELISA. Individual values are shown with the mean of IL-10 concentrations for each condition. *P* values are calculated by two-tailed Student’s t-test (n = 4).
(TIF)

S3 Fig Peripheral blood B cells also secrete IL-10 and display cell surface activation markers following exposure to *L*. *infantum* amastigotes. Purified human B cells isolated from peripheral blood were either left untreated (control) or incubated overnight with *L*. *infantum* amastigotes at a final parasite:host cell ratio of 3:1 (AMA). (A) IL-10 secretion was measured in cell-free supernatants by ELISA. Individual values are shown with the mean of IL-10 concentrations for each condition. *P* values are calculated by two-tailed Student’s t-test (n = 4). (B) Cells and the cell-parasite mixture were washed extensively with a galactose-modified PBS/EDTA solution and stained with anti-C69, anti-CD83 and anti-CD86 antibodies. Samples were read using a BD FACSCanto flow cytometer. Representative histograms depicting CD69, CD83 and CD86 expression are shown in the upper part of the panel. White, light grey, and dark grey histograms represent unstained, control, and AMA-treated, respectively. The lower part of the panel shows the percentages of positive cells and mean fluorescence intensities (MFI) for the indicated cell surface marker. Results represent the mean values of samples from 4 different healthy donors. *P* values are calculated by two-tailed Student’s t-test (n = 4; n.s. = non-significant).
(TIF)
