# Supplementary figures and images for: Leishmania infantum Amastigotes Trigger a Subpopulation of Human B Cells with an Immunoregulatory Phenotype
Source: PLoS Negl Trop Dis. 2015 Feb 24;9(2):e0003543. doi: 10.1371/journal.pntd.0003543 (PMC4339978; doi:10.1371/journal.pntd.0003543)

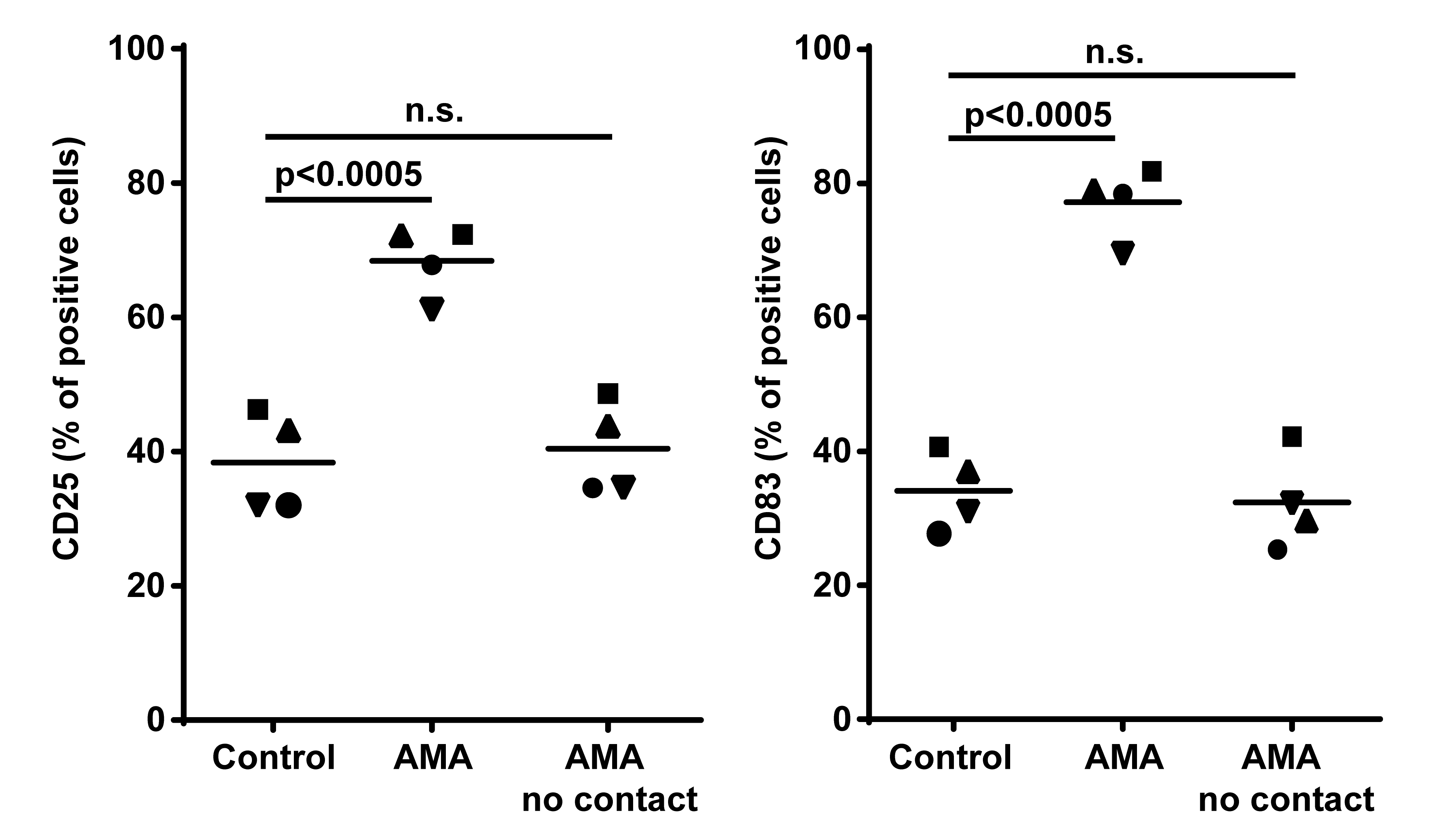

Supplement: S1 Fig — Purified human tonsillar B cells were either left untreated or incubated overnight with L. infantum amastigotes (AMA) at a final parasite:host cell ratio of 3:1. In some wells, cell culture inserts were used to separate parasites and B cells. Cells and the cell-parasite mixture were then intensively washed with a galactose-modified PBS/EDTA solution and stained with anti-CD25 or anti-CD83 antibodies. Samples were read using a BD FACSCanto flow cytometer. Results represent individual and mean values of samples from 4 different healthy donors and are expressed as the percentages of CD25+ (left panel) or CD83+ cells (right panel). P values are calculated by two-tailed Student’s t-test (ns: not significant). (TIF) [file pntd.0003543.s001.tif]

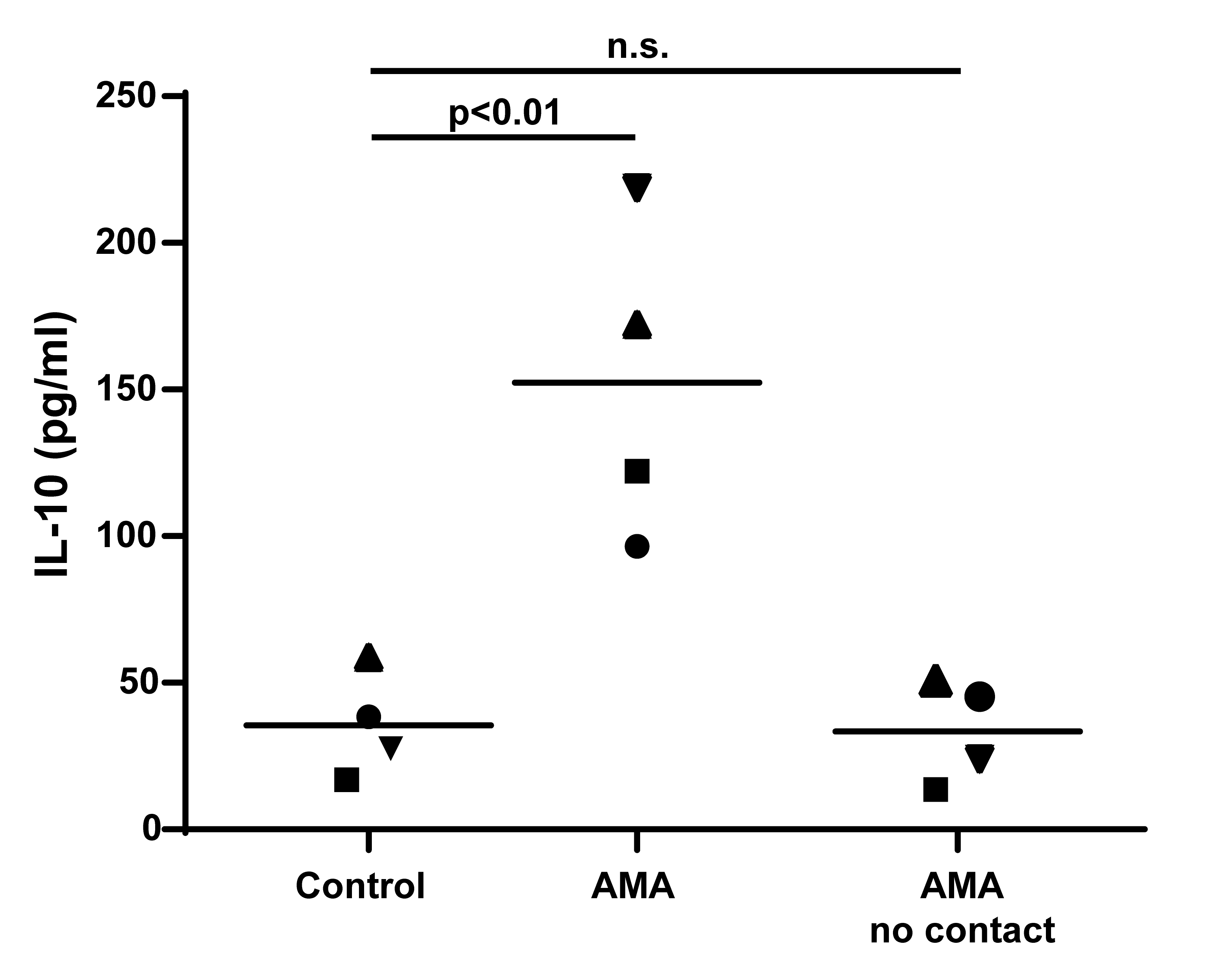

Supplement: S2 Fig — Purified human tonsillar B cells were either left untreated or incubated overnight with L. infantum amastigotes (AMA) at a final parasite:host cell ratio of 3:1. In some wells, cell culture inserts were used to separate parasites and B cells. IL-10 secretion was measured in cell-free supernatants by ELISA. Individual values are shown with the mean of IL-10 concentrations for each condition. P values are calculated by two-tailed Student’s t-test (n = 4). (TIF) [file pntd.0003543.s002.tif]

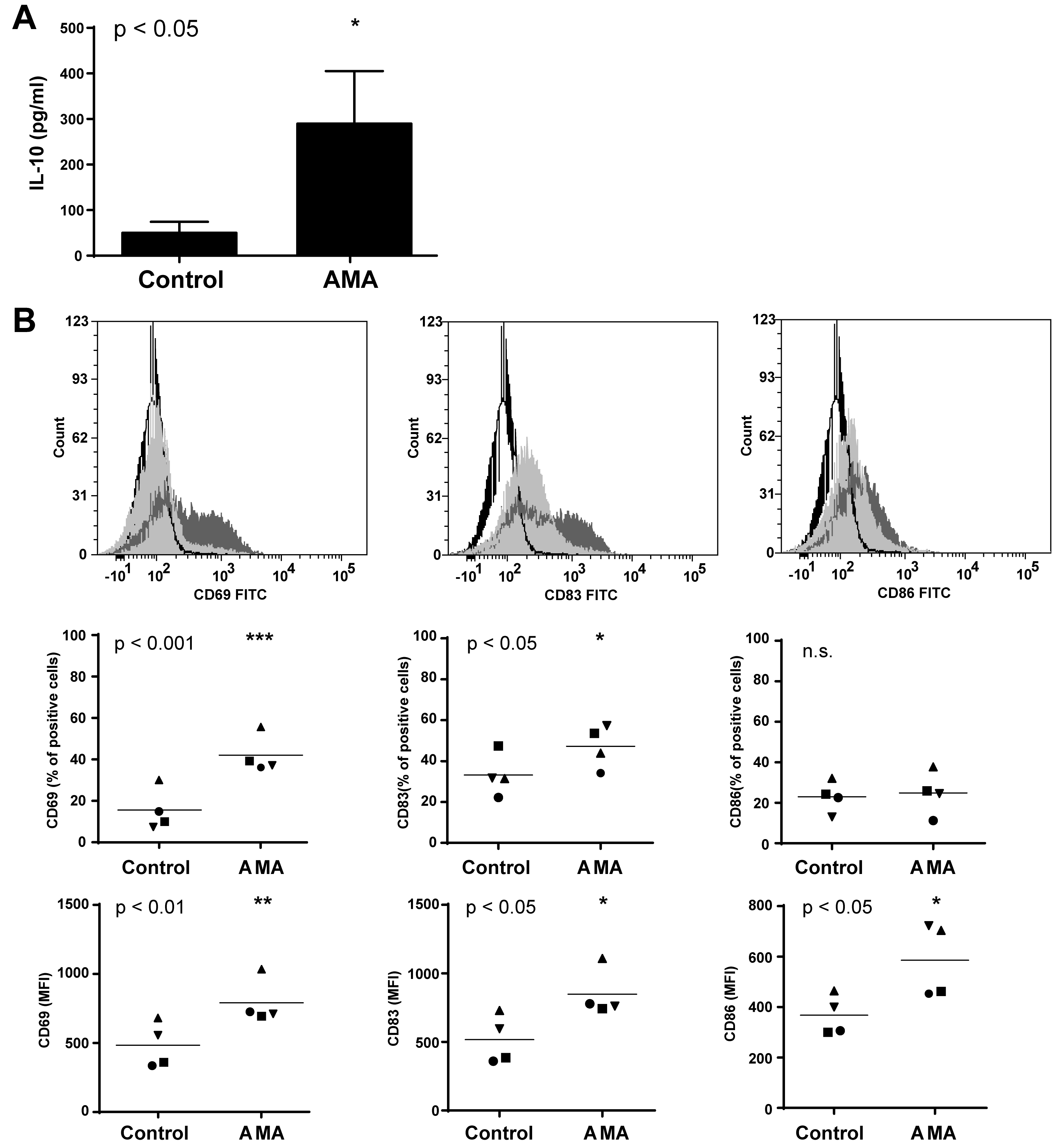

Supplement: S3 Fig — Purified human B cells isolated from peripheral blood were either left untreated (control) or incubated overnight with L. infantum amastigotes at a final parasite:host cell ratio of 3:1 (AMA). (A) IL-10 secretion was measured in cell-free supernatants by ELISA. Individual values are shown with the mean of IL-10 concentrations for each condition. P values are calculated by two-tailed Student’s t-test (n = 4). (B) Cells and the cell-parasite mixture were washed extensively with a galactose-modified PBS/EDTA solution and stained with anti-C69, anti-CD83 and anti-CD86 antibodies. Samples were read using a BD FACSCanto flow cytometer. Representative histograms depicting CD69, CD83 and CD86 expression are shown in the upper part of the panel. White, light grey, and dark grey histograms represent unstained, control, and AMA-treated, respectively. The lower part of the panel shows the percentages of positive cells and mean fluorescence intensities (MFI) for the indicated cell surface marker. Results represent the mean values of samples from 4 different healthy donors. P values are calculated by two-tailed Student’s t-test (n = 4; n.s. = non-significant). (TIF) [file pntd.0003543.s003.tif]
